# Evaluation of expression profiles of APOA4, CEACAM1, CD147, DJ-1/PARK7, Gamma-synuclein, S100A1, and Stathmin-1 in urothelial carcinomas using immunohistochemical assays

**DOI:** 10.3389/fonc.2025.1587558

**Published:** 2025-11-21

**Authors:** Naveed Sharif, Walayat Shah, Asif Ali, Taj Ali Khan, Umar Nishan, Hussah M. Alobaid, Sher Zaman Safi, Syed A. A. Rizv, Nawshad Muhammad

**Affiliations:** 1 Department of Histopathology, Institute of Pathology and Diagnostic Medicine, Khyber Medical University, Peshawar, Khyber Pukhtunkhwa, Pakistan; 2 School of Cancer Sciences, University of Glasgow, Glasgow, United Kingdom; 3 Department of Pathology, College of Medicine, Qassim University, Saudi Arabia; 4 Department of Microbiology, Institute of Pathology and Diagnostic Medicine, Khyber Medical University, Peshawar, Pakistan; 5 Department of Chemistry, Kohat University of Science and Technology, Khyber Pakhtunkhwa, Kohat, Pakistan; 6 Department of Zoology, College of Science, King Saud University, Riyadh, Saudi Arabia; 7 Faculty of Medicine, MAHSA University, Jenjarom, Malaysia; 8 College of Biomedical Sciences Larkin University, Miami, FL, United States; 9 Institute of Basic Medical Science, Khyber Medical University, Peshawar, Khyber Pukhtunkhwa, Pakistan

**Keywords:** bladder cancer, urothelial carcinoma, immunohistochemistry, diagnostic accuracy, gene

## Abstract

**Objective:**

To evaluate the diagnostic performance of APOA4, CEACAM1, CD147, DJ-1/PARK7, Gamma-synuclein, S100A1, and Stathmin-1 in urothelial carcinoma and establish optimal immunohistochemical cutoffs for their use as diagnostic markers.

**Method:**

This cross-sectional study included 141 histologically confirmed urothelial carcinoma cases and controls. Immunohistochemical staining was optimized for each biomarker, and semiquantitative scoring was applied. Diagnostic validity was assessed using receiver operating characteristic (ROC) analysis, comparing sensitivity and specificity across several cutoffs and biomarker panels.

**Results:**

Among seven biomarkers, APOA4, DJ-1/PARK7, Gamma-synuclein, and Stathmin-1 demonstrated high diagnostic accuracy (≥80% sensitivity and specificity). Using an Allred score ≤2 as a cutoff, the sensitivity/specificity were as follows: APOA4, 96%/100%; DJ-1/PARK7, 97%/94%; Gamma-synuclein, 98%/84%; and Stathmin-1, 98%/90%. A combined panel of these four biomarkers achieved near-perfect diagnostic performance, reaching almost 100% sensitivity and specificity.

**Conclusion:**

A biomarker panel comprising Stathmin-1, DJ-1/PARK7, Gamma-synuclein, and APOA4 reliably distinguished urothelial carcinoma from benign urothelium. These markers, when integrated with cytology, could enhance the diagnostic precision and reduce dependence on invasive cystoscopy. The proposed cutoffs (10%–20% positive cells or Allred score ≤2) offer clinically actionable threshold for histopathological practice.

## Introduction

Globally, urothelial carcinoma (UC) is the ninth most frequently identified cancer in both genders (1). UC has four times higher incidence and mortality rates in men as compared to women. The worldwide UC age-standardized incidence rate (ASR) (per 100,000 person/years) is 9.5 for men and 2.4 for women (1, 2). In 2019, a cancer registry-based analysis of data found that UC is the eighth most common cancer among men in Pakistan, with a prevalence rate of 4.8%. It is ranked among the top 5 entities in elderly patients suffering from cancer (3). The risk factors for UC include tobacco smoking; family history; certain occupations like rubber, dye, and textile manufacturing; dietary and environment factors; and infections like schistosomiasis (4). Morphologically, tumors that could invade muscles and soft tissues are known as muscle-invasive bladder cancer (MIBC), and this variety can spread into lymphatics. Approximately 20% of cases of UC belong to this category, while 75% of cases are non-muscle-invasive bladder cancer (NMIBC) (5). A diagnostic imaging method that detects a bladder mass in a patient should be followed by cystoscopy, biopsy, and/or resection to stage and diagnose tissue on histopathological examination (6). Although urine cytology is an effective method for the screening of UC, low cellular yield, urinary tract infections, stone, or intravesical instillations can all interfere with the evaluation of cytology specimens (7). Accurate pathology diagnosis and staging, as well as the full elimination of all noticeable lesions, are the objectives of transurethral resection of bladder tumor (TURBT) in UC cases (8). Currently, urine cytology and cystoscopy with biopsy are the gold standard tests used to detect UC. Cystoscopy is an expensive and invasive procedure, having a risk of infection and overall sensitivity of 62%–84% and specificity of 43%–98% (9). Cytology is an affordable non-invasive urine test that can diagnose and monitor low-grade UC with a maximum sensitivity of 70% and specificity of approximately 99% (10). There is a difference in recurrence rates of NMIBC and MIBC. Almost half of the cases of NMIBC relapse within 5 years of diagnosis and 10% to 15% progress to MIBC (11). In case of MIBC, 50% of cases relapse after radical cystectomy (12). Because of their non-invasiveness, increased precision, and capacity for early identification and recurrence monitoring, biomarkers are completely changing the diagnostic process for UC. Future cystoscopy could be supplemented with trustworthy and patient-friendly biomarker testing because of ongoing research into new biomarkers and their validation.

Several systematic reviews have been published to identify the existing information about the development and utility of IHC biomarkers for UC. These publications also find gaps in existing knowledge about biomarkers for UC. One of the significant systematic reviews was reported by D’Costa et al. (2016) with the objective of classifying these biomarker into four categories: 1) those with well-established sensitivity and specificity for the detection of UC (validated detection biomarkers), 2) those that exhibit promise but need more research (possible biomarkers), 3) those that are unlikely to require additional research (unlikely biomarkers), and 4) those being looked into as prognostic markers. The proteins were classified as “possible biomarkers” with reported sensitivity and specificity rates (sensitivity + specificity/2) ≥80%, which is comparable to the sensitivity and specificity rates of cystoscopy (13). Only the proteins with sensitivity and specificity of at least 80% each were selected for this study project. These include apolipoprotein A4 (APOA4), calprotectin (S100A8/S100A9), carcinoembryonic antigen-related cell adhesion molecule 1 (CEACAM1), CD147, Parkinsonism-associated deglycase (DJ-1or PARK7), Gamma-synuclein, and Stathmin-1 (13).

Although previous studies have explored some of these biomarkers (e.g., DJ-1/PARK7, Stathmin-1, and Gamma-synuclein) primarily through serum- or urine-based ELISA technique, data on their tissue-level expression and immunohistochemical scoring thresholds remain limited. The present study is the first to validate and optimize cutoff values for APOA4, CEACAM1, CD147, DJ-1/PARK7, Gamma-synuclein, S100A1, and Stathmin-1 in a Pakistani population using formalin-fixed paraffin-embedded (FFPE) tissue. Furthermore, by integrating these markers into a composite diagnostic panel, this study introduces a clinically applicable algorithm capable of improving histopathological diagnosis and stratification of UC.

This study aims to determine the expression level and validate the sensitivity and specificity of APOA4, CEACAM1, CD147, DJ-1/PARK7, Gamma-synuclein, S100A1, and Stathmin-1 as diagnostic protein biomarkers in tissue specimens of UC.

## Materials and methods

### Histopathology

The study was held at the Institute of Kidney Diseases Hayatabad Peshawar between January 2020 and December 2022 and involved 141 patients after receiving informed consent and approval from the Institutional Advance Study and Review Board. The institutional ethical committee of Khyber Medical University, Peshawar, provided ethical approval. A non-probability convenience sampling strategy was used in the study to gather participant data. Following established protocols, biopsy and urine samples were collected and sent to the Institute of Pathology and Diagnostic Medicine, Khyber Medical University, Histopathology Laboratory, for additional processing. The two consultant histopathologists and the principal author independently performed a histological investigation on hematoxylin and eosin-stained tissue sections of formalin-fixed paraffin-embedded tumor specimens. The diagnosis was made on the basis of the 2016 WHO categorization (14).

### Immunohistochemistry staining

Following the manufacturer’s recommendations, antibodies were tested by immunohistochemistry (IHC) for APOA4, CD147, CEACAM1, DJ-1/PARK7, Gamma-synuclein, S100A1, and Stathmin-1 on biopsy tissue sections. Details of antibodies with their concentrations and IHC conditions are mentioned in **Table 1**.

**Table 1:** Details of primary antibodies for IHC staining.

Sr. no.	Antibody	Manufacturer	Cat #/ product code	Antigen retrieval solution for HIER*	Antibody dilution	Incubation time	Incubation temperature	Staining location
1.	APOA4	AbClonal Rabbit pAb	A9792/	Citrate buffer pH 6	1/100	60 min	25°C	Cytoplasmic
2.	CEACAM1	Cell Marque Mouse mAb	236M-94/CEA31	Citrate buffer pH 6	1/200	60 min	Room temperature	Cytoplasmic
3.	CD147	Histoline Rabbit mAb	RA0428/BCG/963	Tris EDTA pH 9	1/200	Overnight	4°C	Membranous, cytoplasmic
4.	DJ-1/PARK7	AbClonal Rabbit pAb	A18580/	Citrate buffer pH 6	1/200	Overnight	4°C	Cytoplasmic, membranous
5.	Gamma-synuclein	AbClonal Rabbit pAb	A14492	Citrate buffer pH 6	1/200	60 min	25°C	Cytoplasmic
6.	S100A1	Cell Marque Mouse mAb	330M-14/4C4.9	Citrate buffer pH 6	1/100	60 min	Room temperature	Cytoplasmic, nuclear
7.	Stahtmin-1	AbClonal Rabbit pAb	A2176/	Citrate buffer pH 6	1/400	Overnight	4°C	Cytoplasmic

### Scoring of tissue specimens

The principal author and two consultant histopathologists performed a blinded microscopic analysis of the immunohistochemically stained section slides. Every finding was noted in an Excel spreadsheet. Staining intensity and cell percentage were used to compute a semiquantitative Allred score. The total of these two factors has a range of 0 to 8. Allred scores ranging from 0 to 2 are deemed negative for a given biomarker, whereas scores between 3 and 8 are considered positive (15).

### Statistics and data analysis

Each biomarker’s mean expression in UC was compared to that of non-tumor tissue. The independent sample *t*-test was used to determine statistical significance, and *p*-values were generated. Biomarkers were analyzed for sensitivity and specificity, both individually and in panels, and the results were compared. For this, a receiver operating characteristic (ROC) curve was created. A *p*-value of less than 0.05 was considered statistically significant. We performed statistical analysis using SPSS-23.

## Results

### Clinicopathological features of participants

In our study, the mean age of all the study participants was 61.5 (± 12.2 SD) years, ranging from 27 to 98 years. Out of 91 cases of UC, 74 (81.3%) were equal or less than 70 years of age, and 17 (18.7%) were more than 70 years. UC was more common in men, i.e., 79.1% (72) of the cases, while 20.9% (16) of the cases in the study were women. There were 39 (42.9%) cases with a history of smoking, and 52 (57.1%) cases were non-smokers. All participants presented with hematuria along with obstructive or storage symptoms. All the cases had a mass or tumor in the urinary bladder found on cystoscopy examination with a gross appearance of a papillary pattern. Almost half of the cases have a single tumor (17), while the rest (18) have multiple tumors on cystoscopy examination (**Table 2**).

**Table 2:** Demographic features of the controls and cases of the study.

Characteristics		Controls (*n* = 50)	Cases (*n* = 91)
Age	≤70 years	37 (74%)	74 (81.3%)
	>70 years	13 (26%)	17 (18.7%)
Gender	Female	9 (18%)	19 (20.9%)
	Male	41 (82%)	72 (79.1%)
Smoking	Yes	12 (24%)	39 (42.9%)
	No	38 (76%)	52 (57.1%)
Chief complaints	Hematuria	22 (44%)	71 (78%)
	Hematuria with obstructive symptoms	16 (32%)	6 (6.6%)
	Hematuria with storage symptoms	12 (24%)	14 (15.4%)
Cystoscopy findings	Tumor	0 (0%)	91 (100%)
	Blood clot	25 (50%)	0 (0%)
	Inflammation or redness	25 (50%)	0 (0%)
Tumor status	No tumor	50 (100%)	0 (0%)
	Single tumor	0 (0%)	46 (50.5%)
	Multiple tumor	0 (0%)	45 (49.5%)

There are 38 (41.8%) cases in which the tumor was located on the lateral walls of the UB. Similarly, there were 55 (60.4%) cases of UC with a tumor size equal to or less than 3 cm in diameter. Twenty-six out of 91 cases (28.6%) have atypical cells in urine cytology according to “The Paris System of Reporting Urine Cytology” (19). Other diagnoses included suspicion of high-grade UC (8.8%), high-grade UC (1.1%), and low-grade UC (9.9%). According to the WHO classification of 2016, UC was classified and showed that 40 cases (44%) belong to non-invasive low-grade papillary UC, while 41 cases (45.1%) were found as non-invasive high-grade papillary UC. UC was graded on the two-tier system as per the WHO 2004/2016 and found that 56 cases (61.5%) were high grade and 35 (38.5%) were low grade. The pathological stage of UC was assessed, and it was found that 42 cases (46.2%) belong to pT1 stage, 32 cases (35.2%) have pTa stage, and the rest—17 cases (18.7%)—have the pT2 pathological stage (**Table 3**).

**Table 3:** Pathological features of urothelial carcinoma in the cases of the study.

Features		Number
Site of the urinary bladder	Neck	3 (3.3%)
	Vesicoureteral junction (VUJ)	2 (2.2%)
	Lateral wall	38 (41.8%)
	Anterior wall	8 (8.8%)
	Posterior wall	12 (13.2%)
	Dome	2 (2.2%)
	Multifocal	26 (28.6%)
Size of urothelial carcinoma	≤3 cm	55 (60.4%)
	>3 cm	39 (39.6%)
Cytology diagnosis	Negative for high-grade urothelial carcinoma	47 (51.6%)
	Atypical urothelial cells	26 (28.6%)
	Suspicious of high-grade urothelial carcinoma	8 (8.8%)
	High-grade urothelial carcinoma	1 (1.1%)
	Low-grade urothelial carcinoma	9 (9.9%)
Histopathology diagnosis	Urothelial hyperplasia	1 (1.1%)
	Papillary urothelial neoplasm of low malignant potential	1 (1.1%)
	Non-invasive low-grade papillary urothelial carcinoma	40 (44%)
	Non-invasive high-grade papillary urothelial carcinoma	41 (45.1%)
	Invasive urothelial carcinoma	8 (8.8%)
Histological grade	Low-grade urothelial carcinoma	35 (38.5%)
	High-grade urothelial carcinoma	56 (61.5%)
Pathological stage	pTa	32 (35.2%)
	pT1	42 (46.2%)
	pT2	17 (18.7%)

### Staining characteristics of biomarkers

**Figure 1:**
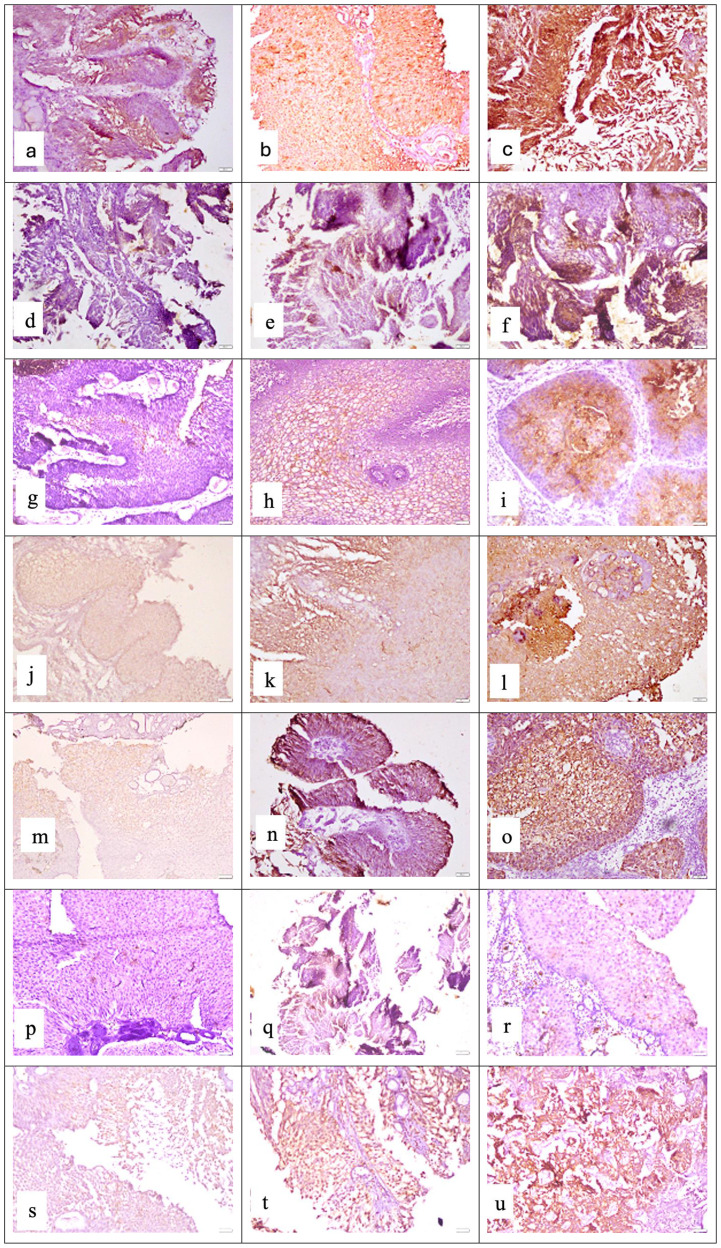
Representative photomicrographs of high-grade urothelial carcinoma showing cytoplasmic staining for APOA4 at **(a)** mild intensity, **(b)** moderate intensity, and **(c)** strong intensity; membranous and cytoplasmic staining for CD147 at **(d)** mild intensity, **(e)** moderate intensity, and **(f)** strong intensity; cytoplasmic staining for CEACAM1 at **(g)** mild intensity, **(h)** moderate intensity, and **(i)** strong intensity; membranous and cytoplasmic staining for DJ-1/PARK7 at **(j)** mild intensity, **(k)** moderate intensity, and **(l)** strong intensity; cytoplasmic staining for Gamma-synuclein at **(m**) mild intensity, **(n)** moderate intensity, and **(o)** strong intensity; nuclear and cytoplasmic staining for S100A1 protein at **(p)** mild intensity, **(q)** moderate intensity, and **(r)** strong intensity; cytoplasmic staining for Stathmin-1 at **(s)** mild intensity, **(t)** moderate intensity, and **(u)** strong intensity. Staining was performed using the horseradish peroxidase (HRP) method with a magnification of ×200.

In UC cases, IHC staining was seen only in epithelial cells for each marker assessment. For each marker assessed in the UC tissue specimens, IHC staining was seen only in epithelial cells (**Table 1**, **Figure 1**).

In general, we observed moderate to strong intensity of staining for APOA4, DJ-1/PARK7, Gamma-synuclein, and Stathmin-1 and negative to mild intensity for CD147, CEACAM1, and S100A1. Moreover, for all four biomarkers with moderate to strong intensity, we found higher expression in tumor cases as compared to normal tissue (normal-looking urothelium in control cases). The mean positive cell expression for biomarkers in cases vs. controls was as follows: for APOA4, 47 vs. 2; for CD147, 10.9 vs. 12.9; for CEACAM1, 9.8 vs. 4.3; for DJ-1/PARK7, 43.6 vs. 7.2; for Gamma-synuclein, 39.5 vs. 4.4; for S100A1, 10.2 vs. 0.6; for Stathmin-1, 37.4 vs. 6.7; for S100A1, 75% vs. 0.3%; for mesothelin, 75% vs. 4%; and for MUC1, 75% vs. 18% (**Table 4**, *p* < 0.0001 for all cases vs. control comparisons except CD147 and CEACAM1).

**Table 4:** Mean positive cell expression of biomarkers in the cases and controls in biopsy.

Biomarker	Controls (mean ± SD)	Cases (mean ± SD)	*p*-value*
APOA4	2 ± 3.1	47 ± 24.5	0.000
CD147	12.9 ± 18.2	10.9 ± 14.3	0.486
CEACAM1	4.3 ± 9.1	9.8 ± 13.6	0.012
DJ-1/PARK7	7.2 ± 10.9	43.6 ± 22.5	0.000
Gamma-synuclein	4.4 ± 6.6	39.5 ± 24.3	0.000
S100A1	0.6 ± 1.2	10.2 ± 15.1	0.000
Stathmin-1	6.7 ± 8.3	37.4 ± 21.0	0.000

*Independent-sample *t*-test.

There was a difference in the mean Allred score of each biomarker in the study between the controls and cases. An Allred score of ≤2 was considered negative, and all control cases showed a mean score <2. The cases of UC showed mean Allred scores of 5.7, 5.4, 5.5, and 5.6 for APOA4, Stathmin-1, DJ-1/PARK7, and Gamma-synuclein, respectively. The mean of Allred scores of CEACAM1 and S100A1 was 2.1 for each. The *p*-value for markers was statistically significant. Only the mean of the Allred score of CD147 for cases was ≤2, i.e., 0.6 (**Table 5**).

**Table 5:** Mean Allred score of biomarkers in the cases and controls.

Biomarker	Controls (mean ± SD)	Cases (mean ± SD)	*p*-value*
APOA4	0.6 ± 0.6	5.7 ± 1.8	0.000
CD147	0.3 ± 0.4	0.6 ± 0.7	0.005
CEACAM1	0.9 ± 1.5	2.1 ± 2.2	0.001
DJ-1/PARK7	1.0 ± 1.3	5.5 ± 1.7	0.000
Gamma-synuclein	0.8 ± 0.9	5.6 ± 1.6	0.000
S100A1	0.5 ± 0.7	2.1 ± 2.0	0.000
Stathmin-1	1.0 ± 1.0	5.4 ± 1.4	0.000

*Independent-sample *t*-test.

### Establishing cutoffs for biomarkers in biopsy

**Figure 2:**
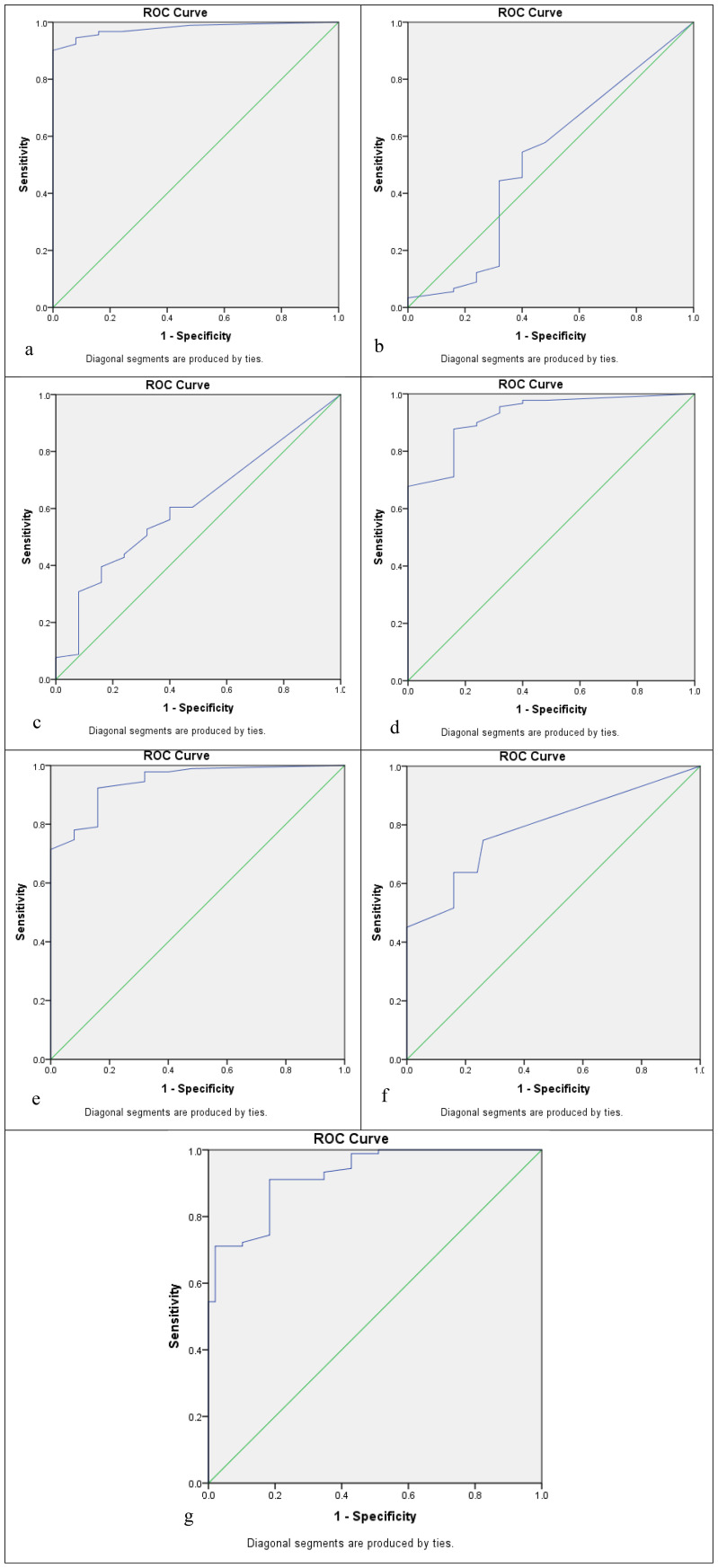
Receiver operating characteristic (ROC) curve analysis to determine the cutoff score for biomarkers: **(a)** APOA4, **(b)** CD147, **(c)** CEACAM1, **(d)** DJ-1/PARK7, **(e)** Gamma-synuclein, **(f)** S100A1, and **(g)** Stathmin-1.

Five cutoffs, or thresholds, for positivity were used to assess the sensitivity and specificity of seven biomarkers. These are as follows: 5% positive cells of any staining intensity (5% cutoff); 10% positive cells of any staining intensity (10% cutoff); 20% positive cells of any staining intensity (20% cutoff); Allred score ≤2; and moderate or strong staining of any cell (+2/+3 cutoff). ROC curve analysis was used to determine three of these cutoffs, which were based on the percentage of positive cells. Each biomarker’s sensitivity was plotted against (1 − specificity), and ROC curves with coordinates were created for each of the seven biomarkers. The area under the curve values in the ROC curve analysis of biomarkers based on the positive percentage of cells for APOA4, CD147, CEACAM1, DJ-1/PARK7, Gamma-synuclein, S100A1, and Stathmin-1 were 0.978, 0.515, 0.612, 0.924, 0.945, 0.789, and 0.925, respectively, as mentioned in **Table 6**, **Figure 2**.

**Table 6:** Area under the curve in the receiver operating characteristic (ROC) curve analysis of biomarkers based on the positive percentage of cells.

Biomarker	Area under the curve	Lower and upper bounds at 95% CI
APOA4	0.978	0.957–0.999
CD147	0.515	0.409–0.621
CEACAM1	0.612	0.518–0.706
DJ-1/PARK7	0.924	0.882–0.967
Gamma-synuclein	0.945	0.911–0.979
S100A1	0.789	0.716–0.862
Stathmin-1	0.925	0.883–0.966

### Sensitivity and specificity of individual biomarkers in biopsy

**Figure 3:**
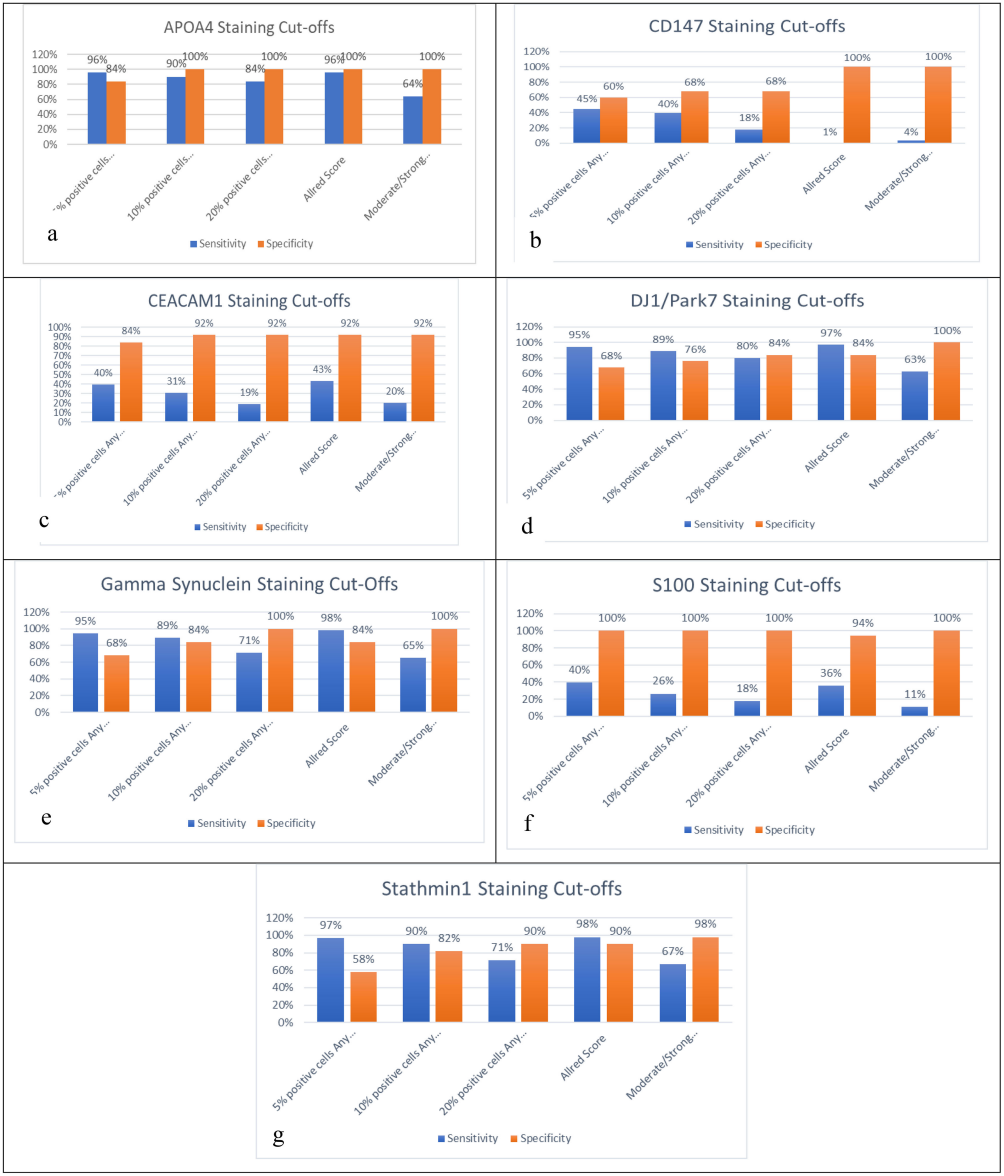
Sensitivity and specificity analysis based on five cutoffs for biomarkers: **(a)** APOA4, **(b)** CD147, **(c)** CEACAM1, **(d)** DJ-1/PARK7, **(e)** Gamma-synuclein, **(f)** S100A1, and **(g)** Stathmin-1.

The sensitivity/specificity of APOA4 was noted as 96%/84%, 90%/100%, 84%/100%, 96%/100%, and 64%/99% for the cutoffs of 5% positive cells, 10% positive cells, 20% positive cells, Allred score ≤2, and moderate or strong staining, respectively. The sensitivity/specificity of CD147 was noted as 45%/62%, 40%/68%, 18%/68%, 1%/100%, and 4%/100% for the cutoffs of 5% positive cells, 10% positive cells, 20% positive cells, Allred score ≤2, and moderate or strong staining, respectively. The sensitivity/specificity of CEACAM1 was noted as 40%/84%, 31%/92%, 19%/92%, 43%/92%, and 20%/92% for the cutoffs of 5% positive cells, 10% positive cells, 20% positive cells, Allred score ≤2, and moderate or strong staining, respectively. The sensitivity/specificity of DJ-1/PARK7 was noted as 95%/68%, 89%/76%, 80%/84%, 97%/84%, and 69%/100% for the cutoffs of 5% positive cells, 10% positive cells, 20% positive cells, Allred score ≤2, and moderate or strong staining, respectively. The sensitivity/specificity of Gamma-synuclein was noted as 95%/68%, 89%/84%, 71%/100%, 98%/84%, and 65%/100% for the cutoffs of 5% positive cells, 10% positive cells, 20% positive cells, Allred score ≤2, and moderate or strong staining, respectively. The sensitivity/specificity of S100A1 was noted as 40%/100%, 26%/100%, 18%/100%, 36%/100%, and 11%/100% for the cutoffs of 5% positive cells, 10% positive cells, 20% positive cells, Allred score ≤2, and moderate or strong staining, respectively. The sensitivity/specificity of Stathmin-1 was noted as 97%/58%, 90%/82%, 71%/90%, 98%/90%, and 62%/98% for the cutoffs of 5% positive cells, 10% positive cells, 20% positive cells, Allred score ≤2, and moderate or strong staining, respectively. APOA4, Stathmin-1, DJ-1/PARK7, and Gamma-synuclein have shown >80% sensitivity and specificity for Allred ≤2 and 10%/20% positive cell cutoffs except moderate/strong staining. The sensitivities and specificities of CD147, CEACAM1, and S100A1 were inconsistent, and none of them showed a sensitivity >80. Hence, they might not be a good diagnostic candidate biomarker when comparing their results between cases and controls (**Figure 3**).

### Sensitivity and specificity of a panel of biomarkers in biopsy

The use of a panel of biomarkers is one method for raising diagnostic sensitivity. For this purpose, the 10% and 20% cutoffs were selected as these two cutoffs provide a fair balance between sensitivity and specificity in the analysis of a single biomarker. Different combinations of panels were tested for their sensitivity and specificity at 10% and 20% positive cells. Each of the panels has >90% sensitivity and specificity. It means that when the four-biomarker panel of APOA4, DJ-1/PARK7, Gamma-synuclein, and Stathmin-1 was considered for the diagnosis of UC in biopsy, it was noted that each had 100% sensitivity and specificity. These findings were similar when any of the promising cutoffs, i.e., 10% positive cells with any intensity, 20% positive cells with any intensity, or ≤2 Allred score cutoff, was applied. Similarly, there were four combinations of the three-biomarker panel of APOAf4, DJ-1/PARK7, Gamma-synuclein, and Stathmin-1. When each of these panels was considered for the cutoffs as mentioned above, each had >98% sensitivity and specificity regarding the diagnosis of UC in biopsy specimens (**Table 7**).

**Table 7:** Panels of biomarkers used for sensitivity and specificity analyses, using 10% and 20% positive cells with any intensity, and ≤2 Allred score as cutoffs for positivity.

Panel of biomarkers	10% positive cells with any intensity as cutoff		20% positive cells with any intensity as cutoff		≤2 Allred score cutoff	
	Sensitivity	Specificity	Sensitivity	Specificity	Sensitivity	Specificity
APOA4, DJ-1/PARK7, Gamma-synuclein, Stathmin-1	100%	100%	100%	100%	100%	100%
APOA4, DJ-1/PARK7, Stathmin-1	100%	100%	99%	100%	100%	100%
APOA4, Gamma-synuclein, Stathmin-1	100%	100%	99%	100%	100%	100%
APOA4, DJ-1/PARK7, Gamma-synuclein	100%	100%	99%	100%	100%	100%
DJ-1/PARK7, Gamma-synuclein, Stathmin-1	100%	99%	98%	100%	100%	100%
APOA4, Stathmin-1	99%	100%	95%	100%	100%	100%
APOA4, DJ-1/PARK7	99%	100%	97%	100%	100%	100%
APOA4, Gamma-synuclein	99%	100%	95%	100%	100%	100%
DJ-1/PARK7, Stathmin-1	99%	96%	94%	98%	100%	100%
Gamma-synuclein, Stathmin-1	99%	97%	92%	100%	100%	100%
DJ-1/PARK7, Gamma-synuclein	99%	96%	94%	100%	100%	100%

## Discussion

UC is one of the common cancers. The non-specific symptoms and late-stage diagnosis contribute to the poor prognosis and survival of patients. It has a remarkably high recurrence rate and is managed according to the grade and stage of the tumor. Despite recent advances in diagnostic modalities, there are still challenges in the timely detection of UC, which ultimately affects the cure and survival rates (20). The current study was designed to determine the expression level and validate the sensitivity and specificity of apolipoprotein A4, S100A1, CD147, CEACAM1, DJ-1/PARK7, Stathmin-1, and Gamma-synuclein as diagnostic protein biomarkers in tissue specimens of UC.

The detailed demographics and clinicopathologic, cytological, and histopathological features were evaluated in this study. In our study, 79.1% of patients were men, which is three times higher than women. Various studies have been published that reported male dominance in cancer prevalence (16, 21, 22), which is consistent with our results. An explanation for the fourfold gender difference in UC incidence could be attributed to men’s greater exposure to tobacco smoke and occupation (23, 24). Gender differences are also caused by differences in sex steroid production and receptor expression (25).

In the present study, the mean age was 61.5 ± 12.2 years at the time of diagnosis of UC. In Western countries, 90% of UC is diagnosed between the age range of 65 to 70 years, and the average age for diagnosis of UC is reported as 73 years (24). However, in Asian populations, UC is diagnosed at a younger mean age, i.e., <65 years, and our findings are consistent with these reports (26). Similarly, 81.3% cases of UC in our study were diagnosed below the 70-year age group.

In our study, all the cases presented with hematuria, and only 22% of cases had other associated symptoms like obstruction- or storage-related urinary symptoms. In fact, hematuria is included as an urgent referral criterion for suspected UC as per NICE guidelines (27).

All cases of UC included in the study had a mass or tumor in the UB with papillary architecture, which was noted during cystoscopy examination. Similar findings were noted by other researchers (28). One of the possible explanations for detecting only papillary architecture in all the tumor cases was the type of cystoscope utilized. In our study, a white light cystoscope was used for all the cases and controls, which is a widely available tool used by urologists with relevant expertise.

As discussed earlier, the number of tumors at the time of diagnosis is a prognostic factor for UC. In our study, there were equal numbers of cases that had either single or multiple tumors at the time of diagnosis of UC. The literature showed variable findings in this regard, and the cases with single tumors are slightly more common, i.e., 57% to 59% (29, 30). The lateral wall was the more common site for the origin of the tumor in our study, and 41.8% of cases had a tumor at this site. A similar finding was reported by a SEER database analysis conducted in 2020 (31). The size of the tumor also has prognostic significance for UC, and we found that the majority of cases, i.e., 60.4%, have a tumor size equal to or less than 3 cm. Identical findings were reported by other investigators (29, 30).

In this study, 61.5% of cases were found as high-grade UC. Several studies have reported similar findings, and the frequency ranged from 60% to 90% (32, 33). We found that the pT1 stage was more common among the UC cases (46.2%). Other researchers also reported almost the same frequency of stage pT1 for UC (34, 35).

In our study, 51.6% of UC cases had urine cytology negative for cancer. A similar frequency was reported by other investigators (36, 37).

It is still troublesome since there is no consistent cutoff for IHC interpretation, nor is there a recognized scoring methodology. Because of this, researchers employ a range of conventional and innovative scoring methods as well as different cutoffs, which may make it difficult for pathologists to accept scoring systems and cutoffs. To completely investigate each of the seven biomarkers’ diagnostic capability, we methodically selected cutoffs from the ROC curve analysis. Pathologists can choose the optimal threshold that is more clinically useful and has the potential to be utilized regularly in pathology by using these cutoffs. This study advances previous biomarker research by providing tissue-based immunohistochemical validation, as opposed to earlier urine or serum ELISA studies that primarily assessed soluble protein levels.

In the current study, the sensitivity and specificity of CD147 in tissue samples were low for all the identified cutoffs. The sensitivity ranged from 45% to 38% and the specificity ranged from 60% to 68% while considering the cutoffs 5%, 10%, and 20%, respectively. CD147 is widely explored for its diagnostic, prognostic, and predictive role for UC, and a higher IHC positive expression is reported according to a semiquantitative method, ranging from 60% to 70% (38, 39). However, the findings of Peng et al. were almost similar to our study (40).

The sensitivity and specificity of CEACAM1 determined in our study for all the cutoff values in tissue specimens were less than 80%. Although Tilki et al. reported 74% and 95% sensitivity and specificity, respectively, for CEACAM1 expression in UC cases, they applied the ELISA method on urine specimens for their analysis (41). In 2015, Soukup et al. also reported a higher sensitivity and specificity for a panel of biomarkers including CEACAM1 for the diagnosis of UC and used the ELISA method on urine samples for an experimental study (42). In contrast to these findings, the loss of expression of CEACAM1 was reported by Ella-Tongwiis et al. in 2020 when they compared the tissue samples of the cases and controls (43). In our study, both the sensitivity and specificity of S100A1 in tissue samples of UC were <80% for all the cutoffs. The underexpression of this biomarker is reported by Yao et al. (44).

Stathmin-1 showed ≥80% for both sensitivity and specificity in tissue specimens for both 10% positive cells and Allred score ≤2 cutoffs. Bhagirath et al. also reported the same sensitivity and specificity for this biomarker by assessing the level in serum and urine via the ELISA method (45). The overexpression of Stathmin-1 in UC was also reported as 68% and 83% by other researchers (18, 46).

In this study, the sensitivity and specificity of DJ-1/PARK7 in tissue specimens were found to be more than 80% at the cutoffs of 20% positive cells and Allred score ≤2. Similar findings were noted by Kumar et al. when they conducted their study on urine samples of UC patients (17). However, Soukup et al. reported lower sensitivity and specificity in urine samples for this marker (47). Lee et al. analyzed the IHC staining of DJ-1/PARK7 in tissue specimens of UC and reported 84% overexpression in them.

Both the sensitivity and specificity of Gamma-synuclein in tissue samples of this study were observed ≥80% in the cutoffs of 10% positive cells and Allred score ≤2. Other researchers reported comparable sensitivity and specificity ranging from 70% to 90% for both, respectively. However, their work was based on conducting the ELISA method on urine samples (48). The overexpression of Gamma-synuclein in tissue samples found by other investigators ranged from 73.5% to 90.7% (49, 50).

In our study, the diagnostic validity of APOA4 in tissue samples was assessed at different cutoffs of 5% positive cells, 10% positive cells, 20% positive cells, and Allred score ≤2, and both sensitivity and specificity were more than 80%. APOA4 is a newer biomarker, and limited findings are available for the validation of this biomarker in the literature. Soukup et al. reported 55.6% sensitivity and 83.3% specificity of APOA4 by utilizing the ELISA technique in urine samples (47). Kumar et al. found that both sensitivity and specificity were more than 80% for this marker (17). By applying a standardized IHC scoring approach and establishing quantitative cutoffs (10%–20% or Allred ≤2), we have improved the translational applicability of these biomarkers in histopathology laboratories.

The lower AUC values for CD147, CEACAM1, and S100A1 may reflect both biological heterogeneity and technical variation. CEACAM1 and CD147 are known to exhibit context-dependent expression, influenced by tumor grade, differentiation, and inflammatory background, which may blur their discriminatory ability in mixed-stage cohorts. For S100A1, cross-reactivity among S100A1 family isoforms could have reduced specificity. Technical factors, such as antibody clone sensitivity and tissue fixation variability, might also have contributed to reduced signal intensity.

A few research studies have reported on the usefulness of biomarker panels, and the majority of IHC diagnostic biomarkers have been studied separately. For examination in a single experimental setting, we carefully chose candidate biomarkers (CD147, CEACAM1, S100A1, Stathmin-1, DJ-1/PARK7, Gamma-synuclein, and APOA4) reported in several studies. We were able to compare these biomarkers and then investigate their diagnostic accuracy in a panel after looking into them in a single cohort. A perfect diagnostic biomarker should be able to distinguish 100% of the disease population (sensitivity) and exclude the normal population (specificity). Since no single biomarker is flawless, a few combinations of these biomarkers were examined to identify the ideal panel for possible therapeutic application. The biomarkers that showed ≥80% for both sensitivity and specificity for most of the cutoffs were chosen for these combinations.

For example, the individual sensitivity/specificity of Stathmin-1 and DJ-1/PARK7 at a cutoff of 20% positive cells was 71%/90% and 80%/84%, respectively. Using a panel of Stathmin-1 and DJ-1/PARK7 improved sensitivity to 94% without much compromising the specificity (98%). Additionally, 100% sensitivity and specificity for both 10% and 20% cutoffs were obtained utilizing a panel comprising Stathmin-1, DJ-1/PARK7, and Gamma-synuclein with at least two positive biomarkers. If two or more biomarkers are positive, this method would place the patient in the tumor-positive category, potentially providing the pathologist with further confidence before placing the patient in the positive group. While developing a panel of biomarkers, researchers have encountered varying degrees of success (51). Notably, the near-perfect diagnostic accuracy achieved by the combined panel (APOA4, DJ-1/PARK7, Gamma-synuclein, Stathmin-1) underscores its superior performance over single-marker assays and its potential as a reliable adjunct to urine cytology in diagnostic workflows.

A wide variety of sensitivity and specificity values for biomarkers may result from the use of various IHC experimental settings. These include primary antibodies, clones, antigen retrieval, antibody dilutions, manual/automated platforms, and other factors by different research groups (52).

We conducted a thorough literature search to find the IHC technique parameters for CD147, CEACAM1, S100A1, Stathmin-1, DJ-1/PARK7, Gamma-synuclein, and APOA4. Prior to staining our cohort, our histology laboratory chose and further optimized those IHC settings that achieved higher diagnostic accuracy.

The comparatively large sample size, the semiquantitative Allred scoring system, the use of numerous biomarker cutoffs, and panel techniques are among the strengths. The primary obstacle to this project’s completion is the scarcity of archived tissues and cytology specimen materials. Finally, we note that our analysis assayed S100A1 by IHC and did not measure calprotectin (S100A8/A9); consequently, our findings should not be generalized to calprotectin without dedicated assays.

## Conclusion

Our findings show that a biomarker panel consisting of Stathmin-1, DJ-1/PARK7, Gamma-synuclein, and APOA4 may accurately diagnose urinary UC in biopsy specimens. This panel, when used in conjunction with cytology, may improve the classification of difficult diagnostic cases in standard clinical practice. From the existing cutoffs, we picked 10%, 20%, moderate staining, strong staining, and Allred score ≤2 cutoffs that have good clinical promise.

## Data Availability

All data generated or analyzed during this study are included in the published article and its supplementary material. Additional information is available from the corresponding author(s) upon request.
